# A Case Report of a Benign Fibrous Histiocytoma: A Post-mosquito Bite Reaction

**DOI:** 10.7759/cureus.39294

**Published:** 2023-05-21

**Authors:** Preethi Annam, Sailaja Nandennagari, Krupavaram Bethala, Reshma Annam, Javairia Ayyub

**Affiliations:** 1 Surgery, Avalon University School of Medicine, Willemstad, CUW; 2 Medical School, Avalon University School of Medicine, Willemstad, CUW; 3 Pharmacology and Therapeutics, Centre of Excellence in Pharmaceutical Sciences, School of Pharmacy, KPJ Healthcare University College, Kuala Lumpur, MYS; 4 Surgery, Windsor University School of Medicine, Cayon, KNA; 5 Surgery, Caribbean Medical University School of Medicine, Willemstad, CUW

**Keywords:** tumor, soft tissue, bony tissue, mosquito bite, fibrous histiocytoma

## Abstract

Fibrous histiocytomas can be differentiated into malignant fibrous histiocytoma or benign fibrous histiocytoma (BFH) and may involve soft tissue and hard bony tissue. A BFH is a rare group of tumors involving fibrocystic and histiocytic elements. Although BFH after a mosquito bite is rare, it must be a part of the differential diagnosis in unresponsive cases of insect bite reactions. A 57-year-old woman with a history of hypertension arrived at the clinic to discuss an 18-year-old lump in her left leg. The patient described the mass as painless and immobile on the anterior part of the left leg. She stated that it had started with a mosquito bite 18 years ago. The woman visited the clinic after her cat scratched the nodule and the erythema on the anterior area of her leg intensified. Physical examination revealed a 10 × 5 × 5 cm solid, stemmed, non-tender mass with erythema on the left leg. A CT scan revealed a stemmed soft tissue mass with well-defined borders. BFH is one of the common types of histiocytoma that is differentiated based on location, which can be dermis, subcutaneous tissue, and deep soft tissues. BFH is predominantly common in young-to-middle-aged females compared to males. BFH can emerge anywhere on the body. However, the occurrence on the lower extremities is high. This case is presented for its rarity and uncommon site of involvement and the significant clinical presentation and emerging condition following a mosquito bite.

## Introduction

A fibrous histiocytoma is a distinctive group of tumors with common fibrocystic and histiocytic elements. Fibrous histiocytoma can be differentiated into malignant fibrous histiocytoma or benign fibrous histiocytoma (BFH) and may involve soft tissue and hard bony tissue [1].

A BFH is a mesenchymal tumor composed of fibroblasts and histiocytes arising in cutaneous and non-cutaneous soft tissues. BFH can grow as a fibrous mass anywhere in the human body, such as the upper or lower extremities, neck, orbit, pelvis, and buccal mucosa [2]. This tumor usually presents as a slow-growing solitary nodule with varied histopathology. BFH most frequently occurs in the dermis compared to subcutaneous and deep soft tissues. The term cutaneous fibrous histiocytoma is usually used to refer to all superficial tumors of the skin regardless of appearance. Similar lesions involving subcutaneous or deep structures are called fibrous histiocytoma [3]. This report presents a rare case of a patient who developed a BFH after a mosquito bite.

## Case presentation

A 57-year-old female with a history of hypertension was bought to the facility for the examination of an 18-year history of a mass in her left leg. The mass was described as painless, pruritic, well-circumscribed, and immobile and was located on the anterior aspect of the left leg. The patient stated that it had started with a mosquito bite 18 years ago. However, the patient visited the clinic when her cat scratched the nodule and the previously present erythema on the anterior part of the leg worsened.

**Figure 1:**
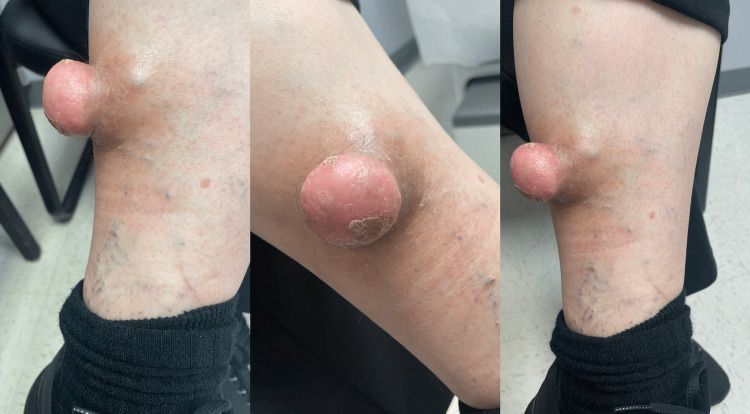
Benign fibrous histiocytoma after a mosquito bite.

Physical examination indicated a firmly stemmed, immovable, well-circumscribed, non-tender mass with erythema on the left leg measuring 10 × 5 × 5 cm. According to the patient, the tumor was initially modest but steadily grew (Figure 1). No regional lymph nodes were palpable. Based on the appearance, differentials analyzed before ordering the labs included dermatofibrosarcoma protuberans (DFSP), malignant fibrous histiocytoma, and solitary fibrous tumor. Routine laboratory workup, including blood count with differential, alkaline phosphatase, and C-reactive protein, was within the normal range except for increased erythrocyte sedimentation rate of 40 mm/hour (normal value = 0-20 mm/hour in women). Chest X-ray, biochemistry, and other blood parameters yielded no pathological finding.

A computed tomography scan revealed a stemmed soft tissue mass with well-defined borders. Incisional biopsy of the mass done under local anesthesia revealed fibroblast and histiocytic infiltrate with mesenchymal origin. Immunohistochemical staining was positive for factor XIIIa and negative for CD34.

An excisional biopsy performed by the surgeon revealed a symmetrical proliferation of histiocytic cells and spindle cells in the dermis on hematoxylin and eosin staining. Excisional biopsy and immunohistochemical studies provided a definitive diagnosis of BFH based on the consistent presence of fibrous tissue containing rounded histiocytes, irregularly arranged spindle cells, and added collagenous and vascular elements of the lesion.

## Discussion

BFHs constitute a group of predominantly benign neoplasms, which exhibit fibroblastic and histiocytic features on histopathology [3]. It is one of the common types of histiocytoma that is differentiated based on location, which can be dermis, subcutaneous tissue, and deep soft tissues. BFH is predominantly common in young-to-middle-aged females compared to males. BFH can emerge anywhere on the body. However, the occurrence on the lower extremities is high [4].

BFHs are characterized by the proliferation of fibroblasts and histiocytes, the presence of foam cells, the absence of cellular atypia, and mitotic changes. Clinically, it can occur as single or multiple well-circumscribed, hyperkeratotic, reddish-brown papules, usually asymptomatic and slow-growing, and measuring 0.5-2.0 cm in diameter. The typical immunohistochemical response pattern of BFH is positive for factor XIIIa and vimentin while negative for CD34, as validated by the spindle tumor cells of BFH in our patient [5].

The histologic variants of this lesion include cellular, epithelioid, aneurysmal, clear cell, lipidized fibrous histiocytoma, which can cause diagnostic difficulty with other benign and malignant tumors, including DFSP, deep fibrous histiocytomas, undifferentiated pleomorphic sarcomas, cutaneous leiomyosarcoma, solitary fibrous tumor, and tenosynovial giant cell tumor [2]. Immunohistochemical staining is essential in differentiating BFH from DFSP, as BFH stains are positive for factor XIIIa and negative for CD34. In contrast, DFSP stains are positive for CD34 and negative for XIIIa [6]. Therefore, a definitive diagnosis of BFH should be made by evaluating the tumor location; radiological characteristics such as the round, soft tissue mass seen in our patient; histopathological features; and immunohistochemical staining results.

The etiology behind the occurrence of BFH after a mosquito bite is unknown. Although BFH is commonly detected in the lower extremities, it is uncommon to be associated with a mosquito bite; nonetheless, we discovered a BFH s/p mosquito bite case in our literature search [7]. Wide surgical excision of the lesion is recommended as a treatment for BFH. Our patient underwent complete local excision of the mass with clear margins without any morbidity. Due to the risk of local recurrence and distant metastases, clinical and radiological follow-ups are highly recommended. Although the use of chemotherapy and radiotherapy is rare, they can be used in the case of distant metastases and multiple recurrences [8]. The incidence of recurrence (Table 1) could be lowered by adjuvant therapy including cryotherapy, which reduces the recurrence rate from 3% to 5%. The differential diagnosis of BFH includes DFSP, deep fibrous histiocytomas, undifferentiated pleomorphic sarcomas, cutaneous leiomyosarcoma, solitary fibrous tumor, tenosynovial giant cell tumor, and Kaposi sarcoma.

**Table 1:** A summary of the characteristics of benign fibrous histiocytoma.

Etiology	Unknown
Incidence	0.1% of all soft tissue tumors
Gender ratio	Slight female predominance
Age predilection	Young to middle-aged (20–50 years)
Risk factors	Unknown
Common sites	Lower extremities
Treatment	Wide surgical excision
Prognosis	Good with low chances of metastasis

## Conclusions

Although the incidence of BFH after a mosquito bite is rare, it can be used as a differential diagnosis in unresponsive cases of insect bite reactions. As these tumors have a wide range of morphology, BFH requires exhaustive histopathological examination and immunohistochemical tests due to their inconclusive baseline investigations. Immunohistochemical staining is extremely useful in differentiating between BFH and DFSP. Complete resection of the lesion is recommended with careful evaluation of the surgical margins. Both benign and malignant fibrous histiocytoma can show local recurrence very rarely and must be treated aggressively.
